# Effects of an integrated supportive program on xerostomia and saliva characteristics in patients with head and neck cancer radiated with a low dose to the major salivary glands: a randomized controlled trial

**DOI:** 10.1186/s12903-022-02225-y

**Published:** 2022-05-23

**Authors:** Nan Jiang, Yue Zhao, Malin Stensson, Jan Mårtensson

**Affiliations:** 1 School of Health and Welfare, Jönköping University, Box 1026, 551 11 Jönköping, Sweden; 2 School of Nursing, Tianjin Medical University, Tianjin, China

**Keywords:** Head and neck cancer, Xerostomia, Unstimulated saliva flow rate, Integrated supportive program

## Abstract

**Background:**

Xerostomia and changes in saliva characteristics are common side-effects in patients with head and neck cancer (HNC) undergoing radiotherapy, which negatively impact their oral health. However, there are no consensus standards for intervention to manage these problems. The aim of this study was to determine the effect of an integrated supportive program on xerostomia and saliva characteristics at a 1-year follow-up of patients with HNC radiated with a low dose to the major salivary glands.

**Methods:**

The CONSORT guidelines for a randomized controlled trial were used. Participants with a low overall dose to major salivary glands were randomly allocated to an intervention group (n = 47) or a control group (n = 45). The intervention group received usual care and an integrated supportive program, which included three steps: face-to-face education; face-to-face coaching at 1 month post-radiotherapy; and four telephone coaching sessions at 2, 3, 6, and 9 months post-radiotherapy. The face-to-face education consisted of oral hygiene instruction, oral self-care strategies, facial and tongue muscle exercises, and salivary gland massage. Adherence to the intervention was evaluated using a questionnaire completed during the 9 months follow-up. The control group received usual care. The unstimulated saliva flow rate and xerostomia were assessed in both groups.

**Results:**

A total of 79 participants (40 in the intervention group and 39 in the control group) completed the 12 months follow-up. The intervention group achieved significantly greater relief from xerostomia than the control group after 3 months (intervention group: 35.1 ± 5.9 versus control group: 38.0 ± 5.9, *P* = 0.027) and 12 months follow-up (intervention group: 18.5 ± 4.1 versus control group: 22.8 ± 4.3, *P* < 0.001). A higher unstimulated saliva flow rate was observed in the intervention group than the control group at 12 months follow-up (intervention group: 0.16 ± 0.08 versus control group: 0.12 ± 0.07, *P* = 0.035). Adherence to the intervention was generally good.

**Conclusion:**

This integrated supportive program with good adherence relieved xerostomia and had a positive effect on unstimulated saliva flow rate among patients with HNC radiated with a low dose to the major salivary glands during the 12 months of follow-up.

*Trial registration*: Chinese Clinical Trial Registry ChiCTR2100051876 (08/10/2021), retrospectively registered.

**Supplementary Information:**

The online version contains supplementary material available at 10.1186/s12903-022-02225-y.

## Background

Head and neck cancer (HNC) is a set of malignancies with a high cancer burden on the healthcare system in China [1]. Treatment for HNC includes external radiotherapy, often in combination with surgery and chemotherapy. The duration of radiotherapy for HNC is six to eight weeks, with a dose of 1.5 ~ 2.0 Grey/day (5 day/week) [2]. Common oral side-effects among patients with HNC during and following radiotherapy are xerostomia (i.e., the subjective feeling of oral dryness) and salivary gland hypofunction. Patients exposed to head and neck irradiation usually have xerostomia or hyposalivation for several months (or years or for their whole life), and this can cause difficulties speaking, chewing, and swallowing and increase the risk for oral disease. Consequently, these problems have a negative impact on oral health and quality of life [3–5].

Several non-pharmacological interventions have been used for the management of xerostomia and hyposalivation; however, the available treatments are generally ineffective and the effect of saliva substitutes is limited [6, 7]. There is no compelling evidence that favours a specific intervention for patients with HNC. Oral hygiene instructions aimed at adults and old adults who have some degree of salivary gland function have shown a positive effect on improving xerostomia [8–11]. Short- or long-term participation in oral functional exercise programs among older adult patients have resulted in improved oral function and fewer problems associated with xerostomia and hyposalivation [12–15]. Furthermore, light massage of the major salivary glands can alleviate xerostomia by increasing blood circulation and parasympathetic activity in older adult patients [15, 16]. However, the target population of these strategies has been older adults who do not have cancer and so have not received radiotherapy.

To our knowledge, there are no consensus standards of intervention for managing radiation-induced xerostomia and changed saliva characteristics among patients with HNC. Accordingly, an integrated supportive program with multicomponent oral care was designed to determine such a program’s effects on the prevalence and severity of xerostomia and saliva characteristics in patients with a diagnosis of HNC radiated with a low dose to the major salivary glands during the first year after radiotherapy.

## Methods

The study was approved by the ethics committee of Tianjin Medical University (TMUHMEC 2,015,008) and the principles of the Declaration of Helsinki were followed. The study was performed in accordance with CONSORT guidelines and registered in the Chinese Clinical Trial Registry ChiCTR2100051876 (08/10/2021), http://www.chictr.org.cn/showproj.aspx?proj=132646.

### Participants

Patients with a confirmed histological diagnosis of HNC were recruited consecutively from one tertiary hospital in Tianjin, China between February 2019 and September 2020. Other eligibility criteria included an age of ≥ 18 years, a Karnofsky performance scale of 80 or more, having primarily received definitive radiotherapy or surgery with postoperative radiotherapy (dose of ≥ 60 grey) with concurrent or induction chemotherapy or both, and the ability to attend for regular re-examination within the 12 months follow-up period. Patients were excluded if they had other cancers, were edentulous, had other causes for xerostomia (i.e., Sjögren syndrome, diabetes mellitus, use of drugs that could interfere with salivary flow, have had bilateral salivary glands surgically removed), or exhibited severe cognitive impairment (i.e., dementia) or psychiatric disorders that interfered with the ability to complete the questionnaire package. Written informed consent was obtained from all participants.

A power analysis determined the sample size. As no previous literature was identified that evaluates the effectiveness of oral care intervention on xerostomia in patients with HNC, we conducted a pre-test study with 15 participants in each group without any significant differences in demographic and disease-related characteristics or xerostomia questionnaire (XQ) score at baseline. The pre-test used the XQ score at the 3 months follow-up to calculate the sample size (mean = 32.7; SD = 5.6) for intervention group and control group (mean = 36.4; SD = 4.9). A sample size of 68 (34 in each group) was required to detect significant differences between the two groups, with a power of 80% and a two-sided 5% level of statistical significance. Ten patients in each group were added to compensate for possible loss to follow-up. The randomization was conducted by a statistician using a simple online binomial randomization program to assign participants to either an intervention group or a control group. At baseline, 47 participants were in the intervention group and 45 participants were in the control group.

### The integrated supportive program

The integrated supportive program was developed by the research group based on evidence from literature reviews [8–17] and the research team’s experience. The program included three steps led by the same researcher (NJ, the first author), a trained coach with experience being a coach in other intervention studies and with rich experience in oncology care for HNC. Details of the program are shown in Table 1.

**Table 1:** Contents of the integrated supportive programme

**Step 1**	**Face-to-face education at baseline at the ward**
Session 1: Oral hygiene instruction	Provide information about the importance of oral hygiene Provide oral hygiene advice (i.e., modified bass teeth brushing method (video), the selection of toothbrush, fluoride toothpaste, and alcohol-free rinses)
Session 2: Self-care instruction	Smoking and drinking alcohol cessation if appropriate Use of mouth-wetting agents Use of sugar-free chewing gum, sucking tablets Frequently sipping water or fluid Intake adequate amount of water/fluid Have fluid food or food with fluid Decrease the frequency of sugar-use Avoid irritating agents Use of air humidifier if appropriate at night
Session 3: Facial and tongue muscle exercise (video)	
Facial muscle	Deep breathes; tightly close eyes and pull lips to both sides of the face (smile) Fully open eyes and mouth Tightly close mouth, fill the mouth with air, and move the air in mouth right and left
Tongue muscle	Extend tongue out far and retract Hold tongue out as far as possible, move it left and right, and move it up and down to lick nose and chin Turn tongue to lick around mouth Push upper and lower lips with tongue Alternately push the left and right cheeks with tongue
Session 4: Salivary gland massage (video)	Check the position of major salivary glands (parotid gland, sublingual gland, and submandibular gland) and massage the glands softly with fingers
**Step 2**	**Face-to-face coaching at outpatient department 1 month post-radiotherapy**
	Nurse coach/participant interactions Listen to participant’s experience of doing programme Assess the adherence of doing programme If adherence was poor or the goals were not being met,explore the possible issues that influenced adherence and discuss solutions Health goals discussion and set a revised plan
**Step 3**	**Telephone coaching at 2, 3, 6, and 9 months post-radiotherapy**
	Nurse coach/participant interactions Listen to participant’s experience with the programme Evaluate the adherence of doing programme Conduct a motivational interview to discuss barriers, possible reasons and solutions Health goals discussion and set a revised plan

Step 1: A researcher (NJ) established trust with the participant and introduced the cause of xerostomia and saliva alteration and their negative effects on oral health and quality of life. The participants received a handbook about the program and viewed a five-minute instruction video about the modified Bass technique of teeth brushing, the muscle exercises, and the salivary gland massage.

Step 2: Face-to-face coaching was conducted by the researcher (NJ) at the outpatient department at 1 month following the completion of radiotherapy. At the end, the researcher collaboratively worked with participants to develop short-term realistic goals. The average time was around 15–20 min.

Step 3: Telephone coaching was performed by the researcher (NJ) 2, 3, 6 and 9 months post-radiotherapy. The researcher kept track of progress of participants’ goals at subsequent telephone coaching sessions. Each telephone coaching session lasted for approximately 15–20 min.

### The usual care

Before radiotherapy, a ward nurse provided usual care, which included face-to-face group-based health education consisting of the following instructions: (1) brush teeth using toothpaste and a soft toothbrush after meals; (2) replace the toothbrush every month; (3) if using dentures, immerse denture in antimicrobial solution for ten minutes; (4) avoid smoking, alcohol, and irritating food; and (5) gargle with medicine (i.e., lidocaine) when experiencing pain due to radiation-induced mucositis. The group-based health education was about 10–15 min and was offered to both the intervention and the control group.

### Data collection

Data on demographic characteristics, xerostomia, saliva characteristics and adherence were collected using questionnaires and tests.

Xerostomia was evaluated using a self-report instrument (the XQ) [18, 19] and clinical oncologists’ assessment (The National Cancer Institute Common Toxicity Criteria for Adverse Events Version 5.0 [CTCAE v5.0]) at baseline, at the end of radiotherapy, and 3 and 12 months post-radiotherapy.

The XQ is an eight-item instrument with four items related to oral dryness while chewing/eating and four items related to oral dryness while not chewing/eating [18]. Each item is scored on a numeric rating scale, ranging from 0 to 10. A total XQ score is calculated by multiplying the sum of the eight items by 1.25 to obtain a final summary score with a range from 0 to 100. Higher scores indicate greater discomfort/dryness [18]. The Chinese version of the XQ, which has excellent validity and reliability, has been used to evaluate xerostomia among patients with HNC in China [19].

The CTCAE v5.0, criteria for standardized classification of adverse effects in cancer treatment, is widely used in the HNC population [20]. The level of xerostomia consists of three grades: Grade 1—symptomatic (e.g., dry or thick saliva) without significant dietary alteration; Grade 2—moderate symptoms with oral intake alterations (e.g., copious water, other lubricants, diet limited to purees and/or soft, moist foods); and Grade 3—inability to adequately feed orally, tube feeding, or total parental nutrition indicated.

Unstimulated whole salivary secretion was measured. The saliva measurements were performed by a PhD student between 8 and 11AM at the clinic. Patients were asked not to eat, drink, brush their teeth or massage their salivary glands for at least two hours before collection. No other conscious movements of the oral musculature were made during collection. Patients were invited to sit down and asked to swallow residual saliva present in the mouth. Next, unstimulated whole saliva samples were collected for 5 min and the secretion rate was expressed in ml/ min. As a minimum volume of saliva was needed, the collection of saliva was continued to 15 min when after 5 min a too low volume was collected. The normal unstimulated saliva flow rate may vary between 0.3 ml/min and 0.4 ml/min; less than 0.1 ml/min is considered to be a very low level.

The adherence Questionnaire included 15 questions based on the content of the program. Participants in the intervention group completed the adherence questionnaire at the outpatient department at the 1 month face-to-face coaching session. A researcher (NJ) assessed the adherence to this questionnaire through telephone interviews at 2, 3, 6 and 9 months coaching sessions.

### Data analysis

SPSS 22.0 (IBM Corp, Armonk, New York) was used to analyse the data. Student’s t-test and the Chi-square test were used to compare baseline data between the two groups. Repeated-measures analysis of variance and independent sample t-test were performed to compare differences in mean XQ scores between the test and control groups. The Mann–Whitney U test was used to compare differences in saliva characteristics between the two groups. *P* < 0.05 was considered statistically significant.

## Results

**Fig. 1:**
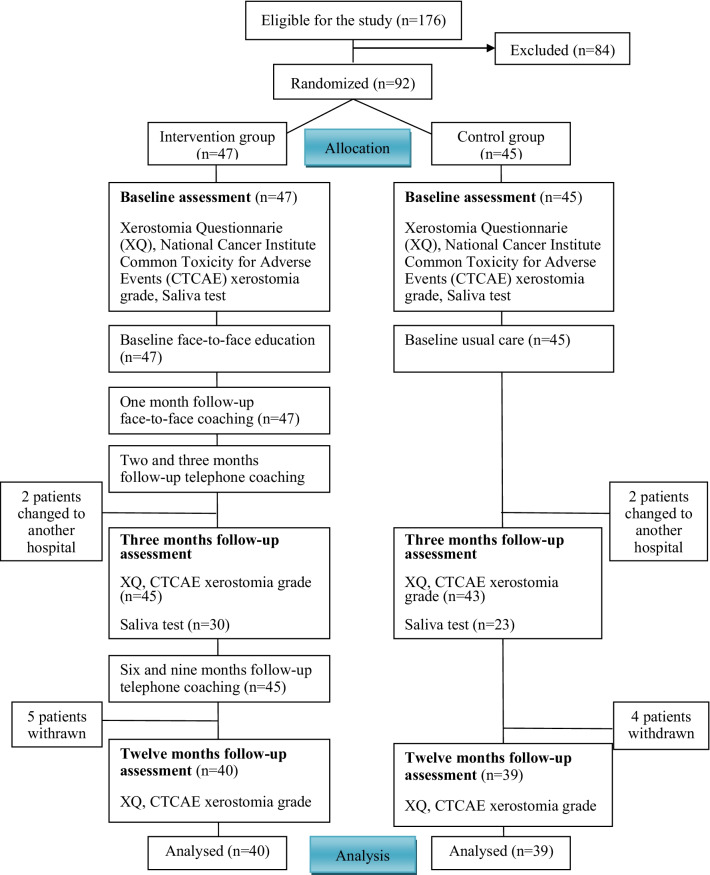
CONSORT flow diagram of participants’ progress through trial phases

A total of 92 participants were enrolled, and 79 (86%) completed the 12 months follow-up (Fig. 1).

The lost 13 patients were somewhat older than the 79 patients who completed the 12 months follow-up (mean age 55 and 49 year) and differ with regard to baseline characteristics (marital status, smoking habits. alcohol consumption, economic situation and working status) (Additional file 1: Table S1).

Characteristics of the participants of the study sample are shown in Table 2. The mean radiation dose to the parotid gland were 27.2 Gy for intervention group and 28.9 Gy for control group and the mean radiation dose to the submandibular glands were 30.9 Gy for intervention group and 33.3 Gy for control group. There were no significant differences in demographic or disease-related characteristics at baseline between the two groups.

**Table 2:** Demographic and disease-related characteristics of the participants, n (%) (n = 92)

Characteristics		Intervention group (n = 47)	Control group (n = 45)
Age (years), mean ± SD		48.1 ± 10.8	51.9 ± 10.9
Body weight (kg), mean ± SD		73.7 ± 10.4	72.4 ± 11.7
BMI, mean ± SD		25.2 ± 3.3	25.3 ± 2.4
Sex, n (%)	Male	38 (80.9)	33 (73.3)
	Female	9 (19.1)	12 (26.7)
Karnofsky performance status (score), n (%)	80	14 (29.8)	12 (26.7)
	90	32 (68.1)	30 (66.7)
	100	1 (2.1)	3 (6.6)
Marital status, n (%)	Married/cohabitant	41 (87.2)	35 (77.8)
	Widowed/Divorced/Single	6 (12.8)	10 (22.2)
Education level, n (%)	Primary or below	8 (17.0)	7 (15.6)
	Secondary	23 (48.9)	26 (57.8)
	Tertiary	16 (34.1)	12 (26.6)
Smoking, n (%)	Never smoker	6 (12.8)	11 (24.4)
	Current smoker	20 (42.6)	13 (28.9)
	Ex-smoker	21 (44.6)	21 (46.7)
Alcohol consumption, n (%)	None, n (%)	29 (61.7)	34 (75.6)
	Drinks > 1 standard glass per week	18 (38.3)	11 (24.4)
Economic situation, n (%)	Very good	4 (8.6)	3 (6.7)
	Good	19 (40.4)	20 (44.4)
	Problematic	19 (42.4)	18 (40.0)
	Very problematic	5 (10.6)	4 (8.9)
Living arrangement, n (%)	Living with partner	43 (91.5)	39 (86.7)
	Living alone	4 (8.5)	6 (13.3)
Working status, n (%)	Working	17 (36.2)	15 (33.3)
	On sick-leave	15 (31.9)	14 (31.2)
	Unemployed	9 (19.1)	10 (22.2)
	Retired	6 (12.8)	6 (13.3)
Physical exercise, n (%)	Inactive	32 (68.1)	28 (62.2)
	At least 30 min per day	15 (31.9)	17 (37.8)
Tumour site, n (%)	Nasopharynx	20 (42.6)	17 (37.8)
	Larynx	7 (14.8)	6 (13.3)
	Hypopharynx	6 (12.8)	3 (6.7)
	Oropharynx	6 (12.8)	8 (17.8)
	Oral cavity	8 (17.0)	11 (24.4)
Tumour node metastasis staging system, n (%)	II	8 (17.0)	8 (17.8)
	III	29 (61.7)	22 (48.9)
	IV	10 (21.3)	15 (33.3)
Treatment modality, n (%)	Radiotherapy and chemotherapy	25 (53.2)	25 (55.6)
	Radiotherapy, chemotherapy and surgery	18 (38.3)	16 (35.5)
	Radiotherapy	4 (8.5)	4 (8.9)
Radiation dose to the parotid gland (Gy), mean ± SD		27.2 ± 12.0	28.9 ± 11.3
Radiation dose to the submandibular glands (Gy), mean ± SD		30.9 ± 15.8	33.3 ± 14.2

A significant increase in the mean score of the XQ was observed at the end of radiotherapy, and the mean scores for the XQ had decreased at the 3 and 12 months follow-up in both groups (Table 3). The interaction effect between time and group showed that the intervention group recovered significantly better than the control group in XQ (*P* = 0.049). The difference of XQ between intervention and control groups was statistically significant at the 3 and 12 months follow-up (*P* = 0.027 and *P* < 0.001, respectively).

**Table 3:** Longitudinal changes and significant differences of XQ in intervention and control groups over the 12 months follow-up (n = 79)

Group	Baseline	End of radiotherapy	Three months post- radiotherapy	Twelve months post- radiotherapy	F time*group	*P*^*a*^
Intervention group	12.7 ± 3.5	55.2 ± 9.4	35.1 ± 5.9	18.5 ± 4.1	3.062	0.049
Control group	12.4 ± 3.2	55.8 ± 8.3	38.0 ± 5.9	22.8 ± 4.3		
t	0.420	− 0.293	− 2.254	− 4.453		
*P*^*b*^	.676	.770	.027	< .001		

*P*^a^ indicates the result of the repeated-measures analysis of variance to determine the differences in the mean score of XQ between the intervention and control groups over time

*P*^b^ indicate the results of the independent sample t test to determine the differences in the mean score of XQ between the intervention and control groups

Around 90% of patients in both groups reported CTCAE grade 0 xerostomia at baseline (Table 4). At the 12 months follow-up, the number of grade 2 participants had decreased in both groups. Grade 3 was only experienced at the end of radiotherapy. There were no significant differences between the two groups in grade level of xerostomia at baseline, at the end of radiotherapy, or after 3 months, but the grade of xerostomia was significantly lower in the intervention group at the 12 months follow-up (*P* = 0.046).

**Table 4:** The effects of an integrated supportive programme on CTCAE, n (%) (n = 79)

Variable	Group	CTCAE				*X*^*2*^	*P*
		Grade 0	Grade 1	Grade 2	Grade 3		
Baseline	Intervention group	37 (92.5)	3 (7.5)	–	–	0.186	0.666
	Control group	35 (89.7)	4 (10.3)	–	–		
End of radiotherapy	Intervention group	–	–	32 (80.0)	8 (20.0)	0.288	0.591
	Control group	–	–	33 (84.6)	6 (15.4)		
Three months post- radiotherapy	Intervention group	–	14 (35.0)	26 (65.0)	–	2.942	0.086
	Control group	–	7 (17.9)	32 (82.1)	–		
Twelve month post- radiotherapy	Intervention group	7 (17.5)	23 (57.5)	10 (25.0)	–	6.176	0.046
	Control group	6 (15.4)	13 (33.3)	20 (51.3)	–		

CTCAE, Common Terminology Criteria for Adverse Events; Grade 0, no xerostomia; Grade 1, symptomatic (e.g., dry or thick saliva) without significant dietary alteration; Grade 2, moderate symptoms, oral intake alterations (e.g., copious water, other lubricants, diet limited to purees and/or soft, moist foods); Grade 3, inability to adequately aliment orally, tube feeding, or total parental nutrition indicated

There was a significant reduction in unstimulated saliva flow after 3 months in both groups, followed by a recovery after 12 months (Table 5). The mean unstimulated saliva flow did not return to baseline after 12 months in either group. However, a statistically significant difference between the groups was found after 12 months (*P* = 0.035) with better recovery in the intervention group.

**Table 5:** The effects of an integrated supportive programme on saliva characteristics (n = 79)

Saliva characteristics	Follow-up time	Group	Mean ± SD	Z	*P*
Unstimulated flow rate, ml/min	Baseline	Intervention group	0.48 ± 0.12	− 0.271	0.786
		Control group	0.49 ± 0.10		
	Three months post- radiotherapy	Intervention group ^a^	0.06 ± 0.04	− 0.797	0.425
		Control group ^b^	0.05 ± 0.05		
	Twelve months post- radiotherapy	Intervention group	0.16 ± 0.08	− 2.111	0.035
		Control group	0.12 ± 0.07		

^a^30 saliva samples for analysis in intervention group at 3 months post- radiotherapy

^b^23 saliva samples for analysis in control group at 3 months post- radiotherapy T

The adherence to the intervention was generally good, especially for the first 3 months, with a slight decreasing after 3 months. High adherence was seen for frequently sipping water/fluid and decreasing their consumption of sweet foods and drinks. Lowest adherence was found with the use of mouth-wetting agents, sugar-free chewing gum and sucking tablets (Additional file 1: Table S2).

## Discussion

This study aimed to evaluate the effects of an integrated supportive program on xerostomia and saliva characteristics among patients with HNC radiated with a low dose to the major salivary glands. The program significantly relieved xerostomia and improved unstimulated saliva flow rate in those patients during the 12 months.

There are some weaknesses of the study design and measures that needed to be considered. First, this study was conducted at one tertiary hospital with a relatively small sample size. This large hospital receives patients with cancer from all over the country and nearly 500 patients with HNC are admitted annually. However, to ensure that patients could complete all follow-up visits, only patients who participated in regular re-examinations were included in this study; this might have led to a degree of selection bias. Future research should explore whether these findings can be replicated in a large multicenter context with a larger sample. Second, patients with a Karnofsky performance scale of 80 or more were included in this study. The scale ratings ranging from 80 to 100 reflect the clinical status of person who is able to carry out normal activities (e.g., work) with or without effort. Patients with a score of 80 to 100 were in a better performance status during and after radiotherapy and so were probably more able and willing to complete a long-term intervention and follow-up. This may have led to better adherence and less data loss in this intervention. However, this might also compromise the generalizability of the findings. Third, unstimulated saliva flow rate was assessed in the study, but stimulated saliva flow rate was not. One reason for this was that, even if stimulated saliva is more commonly measured, unstimulated saliva is likely more representative of a patient’s daily symptoms and best reflects basic saliva production. The output of unstimulated saliva was measured volumetrically in this study. However, since this method may have measurement errors because of the bubbles, weight measurement is better recommended in patients with low salivary secretion [21]. Duplicating measurement at different time-points is also recommended, because it can produce more reliable data. Considering shortening the time of data collection, most of patients spent 5 min to collect saliva and only those patients with unstimulated flow rate less than 0.1 ml/min spent 15 min to collect it. This allows patients to have various collection times, which may make it difficult to compare results. Therefore, it is recommended to use a uniform saliva collection time to facilitate comparison of results in future studies. Fourth, in this study, patients with an overall low dose to the major salivary glands were selected, as these patients’ salivary flow usually recovers to some extent within 12 months. The 1-year follow-up period may be too short to determine the effect of an integrated supportive program on salivary secretion and xerostomia in patients with high radiation dose to salivary glands as these patients usually have severe damage to salivary gland function which may need several years to recover (Jensen et al., 2010). Thus, using a longer follow-up for patients with high radiation dose to salivary glands is suggested for further research. Fifth, the researcher (NJ) did the intervention and assessed the adherence to the intervention, introducing a potential bias. This arrangement was necessary because no other researchers/clinicians with the necessary qualifications and experience to train participants were available. In fact, there are few oncology nurses with the necessary coaching qualifications in China. Accordingly, more nurses will need to be trained if similar follow-up interventions are to be tested. Lastly, there was a risk of contamination or dissemination of the first step of intervention to the control group since the participants were inpatients in the same hospital. However, contamination or dissemination of the second and third step intervention was unlikely because patients were outpatients with little risk of meeting again. Despite the above weaknesses, this study is the first randomized control trial with a multicomponent oral care intervention for patients with HNC and so it adds valuable knowledge within this highly important research area.

Interestingly, a significantly better relief of xerostomia and higher mean value for unstimulated saliva flow rate were observed in the intervention group at the 12-month follow-up than in the control group. This finding might be questioned, since radiotherapy can affect salivary glands so much that they take a long time to recover or do not recover at all. The level of salivary gland hyposalivation depends on differential damage of glands as the result of different irradiation volumes and doses [22]. However, there was no difference between the intervention and control groups in the mean radiation dose to the parotid gland and submandibular glands. This suggests that this program had positive long-term effects on unstimulated saliva flow. Therefore, nurses should motivate patients to prioritize oral hygiene strategies, oral function exercises, and salivary gland massage as an integral part of oncology care before radiotherapy, and then continue that for several years after follow-up or until a patient’s oral health status has largely recovered.

Adherence has been identified as a prerequisite for good outcomes. The adherence to this program was generally good, but decreased slightly after the 3 months follow-up. To increase adherence, one suggestion is to extend the follow-up time to 5 years post-radiotherapy (i.e., a brief coaching intervention every 3 or 6 months for the first 5 years). This may be possible to implement in China, where patients with HNC regularly visit a physician in the clinic for re-examination to check for reoccurrence of cancer within 5 years after cancer treatment. Another suggestion is to train physicians and outpatient nurses in the coaching intervention, even though this might increase their workload. That workload might be reduced by using mHealth, a new model of remote health delivery via mobile phone, which is increasingly used in China and has proved to be a useful way to improve adherence to care instructions in patients with chronic conditions [23]. It would be interesting to test adherence to this program using an mHealth approach. For example, mHealth could be used to provide weekly text message reminders about health care instructions post-radiotherapy. In addition, in future research, a healthcare professional might provide personalized monthly counseling and feedback through mHealth.

## Conclusions

An integrated supportive program with good adherence was effective at relieving xerostomia and increasing unstimulated saliva flow rate among patients with HNC radiated with a low dose to the major salivary glands during a 12 months follow-up post-radiotherapy. Further study is needed to refine this intervention and evaluate the effects of the intervention on the stimulated flow rate.

## Supplementary Information

**Additional file 1. Supplementary table **1. Comparison of baseline characteristics of the 79 patients who completed thetwelve months follow-up and the 13 patients that didn’t complete the study.** Supplementary table 2**. Adherenceto intervention for intervention group.

## Data Availability

The data generated in this study was available from Chinese Clinical Trial Registry website (http://www.chictr.org.cn/showproj.aspx?proj=132646).

## Abbreviations

CTCAE: The National Cancer Institute Common Toxicity Criteria for Adverse Events
HNC: Head and neck cancer
XQ: Xerostomia questionnaire
