# Supplementary material for: Effects of an integrated supportive program on xerostomia and saliva characteristics in patients with head and neck cancer radiated with a low dose to the major salivary glands: a randomized controlled trial
Source: BMC Oral Health. 2022 May 23;22:199. doi: 10.1186/s12903-022-02225-y (PMC9125898; doi:10.1186/s12903-022-02225-y)
Supplement: Supplementary file 1 — Additional file 1. Supplementary table 1. Comparison of baseline characteristics of the 79 patients who completed thetwelve months follow-up and the 13 patients that didn’t complete the study. Supplementary table 2. Adherenceto intervention for intervention group. [file 12903_2022_2225_MOESM1_ESM.docx]

**Supplementary table 1** Comparison of baseline characteristics of the 79 patients who completed the twelve months follow-up and the 13 patients that didn’t complete the study

| Characteristics |  | Group A  (n=13) | Group B  (n=79) | t/X^2^ |
| --- | --- | --- | --- | --- |
| Age (years), mean ±SD | | 55.5±11.2 | 49.1±10.7 | 1.983**^*^** |
| Body weight (kg), mean ±SD | | 72.7±9.6 | 73.2±11.2 | -0.131 |
| BMI, mean ±SD | | 24.3±2.3 | 25.4±2.9 | -1.337 |
| Sex, n (%) | Male | 11 (84.6) | 60 (75.9) | 0.476 |
|  | Female | 2 (15.4) | 19 (24.1) |  |
| Karnofsky performance status (score), n (%) | 80 | 5 (38.5) | 21 (26.6) | 1.293 |
|  | 90 | 8 (61.5) | 54 (68.4) |  |
|  | 100 | 0 (0) | 4 (5.1) |  |
| Marital status, n (%) | Married/cohabitant | 8 (61.5) | 68 (86.1) | 4.678**^*^** |
|  | Widowed/Divorced/Single | 5 (38.5) | 11 (13.9) |  |
| Education level, n (%) | Primary or below | 5 (38.5) | 10 (12.7) | 5.829 |
|  | Secondary | 6 (46.1) | 43 (54.4) |  |
|  | Tertiary | 2 (15.4) | 26 (32.9) |  |
| Smoking, n (%) | Never smoker | 4 (30.8) | 13 (16.4) | 8.512**^*^** |
|  | Current smoker | 0 (0) | 33 (41.8) |  |
|  | Ex-smoker | 9 (69.2) | 33 (41.8) |  |
| Alcohol consumption, n (%) | None, n (%) | 13 (100) | 50 (63.3) | 6.969**^**^** |
|  | Drinks > 1 standard glass per week | 0 (0) | 29 (36.7) |  |
| Economic situation, n (%) | Very good | 0 (0) | 7 (8.9) | 8.462**^*^** |
|  | Good | 4 (30.8) | 35 (44.3) |  |
|  | Problematic | 5 (38.4) | 32 (40.5) |  |
|  | Very problematic | 4 (30.8) | 5 (6.2) |  |
| Living arrangement, n (%) | Living with partner | 12 (92.3) | 70 (88.6) | 0.158 |
|  | Living alone | 1 (7.7) | 9 (11.4) |  |
| Working status, n (%) | Working | 0 (0) | 32 (40.5) | 10.676**^*^** |
|  | On sick-leave | 8 (61.5) | 21 (26.6) |  |
|  | Unemployed | 4 (30.8) | 15 (19.0) |  |
|  | Retired | 1 (7.7) | 11 (13.9) |  |
| Physical exercise, n (%) | Inactive | 11 (84.6) | 49 (62) | 2.511 |
|  | At least 30 minutes per day | 2 (15.4) | 30 (38) |  |
| Tumour site, n (%) | Nasopharynx | 7 (53.8) | 30 (38) | 3.709 |
|  | Larynx | 2 (15.4) | 11 (13.9) |  |
|  | Hypopharynx | 2 (15.4) | 7 (8.9) |  |
|  | Oropharynx | 0 (0) | 14 (17.7) |  |
|  | Oral cavity | 2 (15.4) | 17 (21.5) |  |
| tumour node metastasis staging system, n (%) | Ⅱ | 3 (23.0) | 13 (16.5) | 1.778 |
|  | Ⅲ | 5 (38.5) | 46 (58.2) |  |
|  | Ⅳ | 5 (38.5) | 20 (25.3) |  |
| Treatment modality, n (%) | Radiotherapy and chemotherapy | 7 (53.8) | 43 (54.4) | 0.936 |
|  | Radiotherapy, chemotherapy and surgery | 4 (30.8) | 30 (38) |  |
|  | Radiotherapy | 2 (15.4) | 6 (7.6) |  |

**Group A**: the 13 patients that didn’t complete the study; **Group B**: the 79 patients who completed the twelve months follow-up

**^*^P<0.05; ^**^P<0.01**

**Supplementary table 2** Adherence to intervention for intervention group n (%) (n=40)

| Question |  | One month | Two month | Three month | Six month | Nine month |
| --- | --- | --- | --- | --- | --- | --- |
| Q1 | How often did you brush your teeth last month? |  |  |  |  |  |
|  | More than twice a day | 29 (72.5) | 31 (77.5) | 28 (70) | 26 (65) | 21 (52.5) |
|  | Twice a day | 11 (27.5) | 9 (22.5) | 12 (30) | 14 (35) | 19 (47.5) |
|  | Once a day | - | - | - | - | - |
|  | 4–6 times a week | - | - | - | - | - |
|  | 1–3 times a week | - | - | - | - | - |
|  | 1–3 times a month | - | - | - | - | - |
|  | Never | - | - | - | - | - |
| Q2 | Do you use a toothpaste that contains fluoride? |  |  |  |  |  |
|  | Always (50% or more than) | 33 (82.5) | 4 (100) | 40 (100) | 40 (100) | 40 (100) |
|  | Often (less than 50%) | - | - | - | - | - |
|  | Never | - | - | - | - | - |
|  | Don’t know | 7 (17.5) | - | - | - | - |
| Q3 | Do you use alcohol-free rinses? |  |  |  |  |  |
|  | Always (50% or more than) | 23 (57.5) | 24 (60) | 27 (67.5) | 23 (57.5) | 20 (50) |
|  | Often (less than 50%) | 17 (42.5) | 15 (37.5) | 12 (30) | 11 (27.5) | 12 (30) |
|  | Never | - | 1 (2.5） | 1(2.5) | 6 (15) | 8 (20) |
| Q4 | Do you do facial muscle exercises? If yes, how often? |  |  |  |  |  |
|  | Three times or more a day | 14 (35) | 13 (32.5) | 16 (40) | 13 (32.5) | 9 (22.5)- |
|  | Once or twice a day | 12 (30) | 13 (32.5) | 9 (22.5) | 4 (10) | 6 (15) |
|  | 4–6 times a week | 10 (25) | 10 (25) | 7 (17.5) | 10 (25) | 10 (25) |
|  | 1–3 times a week | 2 (5) | 4 (10) | 8 (20) | 13 (32.5) | 15 (37.5) |
|  | 1–3 times a month | - | - | - | - | - |
|  | Never | - | - | - | - | - |
| Q5 | Do you do tongue muscle exercises? If yes, how often? |  |  |  |  |  |
|  | Three times or more a day | 13 (32.5) | 13 (32.5) | 14 (35) | 13 (32.5) | 10 (25) |
|  | Once or twice a day | 13 (32.5) | 12 (30) | 9 (22.5) | 3 (7.5) | 7 (17.5) |
|  | 4–6 times a week | 7 (17.5) | 14 (35) | 11 (27.5) | 15 (37.5) | 15 (37.5) |
|  | 1–3 times a week | 7 (17.5) | 2 (5) | 6 (15) | 9 (22.5) | 8 (20) |
|  | 1–3 times a month | - | - | - | - | - |
|  | Never | - | - | - | - | - |
| Q6 | Do you do salivary gland massage? If yes, how often? |  |  |  |  |  |
|  | Three times or more a day | 21 (52.5) | 26 (65) | 22 (55) | 20 (50) | 21 (52.5) |
|  | Once or twice a day | 10 (25) | 9 (22.5) | 9 (22.5) | 9 (22.5) | 4 (10) |
|  | 4–6 times a week | 8 (20) | 4 (10) | 9 (22.5) | 8 (20) | 11 (27.5) |
|  | 1­–3 times a week | 1 (2.5) | 1 (2.5) | - | 3 (7.5) | 4 (10) |
|  | 1–3 times a month | - | - | - | - | - |
|  | Never | - | - | - | - | - |
| Q7 | Do you smoke? |  |  |  |  |  |
|  | I have never been a smoker | 5 (12.5) | 5 (12.5) | 5 (12.5) | 5 (12.5) | 5 (12.5) |
|  | I stopped smoking more than 6 months ago | 11 (27.5) | 13 (32.5) | 14 (35) | 26 (65) | 27 (67.5) |
|  | I stopped smoking less than 6 months ago | 14 (35) | 14 (35) | 14 (35) | 3 (7.5) | 3 (7.5) |
|  | I smoke, but not daily | 3 (7.5) | 2 (5) | 1 (2.5) | 1 (2.5) | 1 (2.5) |
|  | I smoke daily | 7 (17.5) | 6 (15) | 6 (15) | 5 (12.5) | 4 (10) |
| Q8 | If a smoker, how many cigarette packages per week? |  |  |  |  |  |
|  | Non-smoker | 30 (75) | 32 (80) | 33 (82.5) | 34 (85) | 35 (87.5) |
|  | 1–3 packages | 6 (15) | 6 (15) | 6 (15) | 6 (15) | 5 (12.5) |
|  | 4–6 packages | 4 (10) | 2 (5) | 1 (2.5) | - | - |
| Q9 | How many standard glasses of alcohol do you drink per week? |  |  |  |  |  |
|  | None | 40 (100) | 40 (100) | 37 (92.5) | 34 (85) | 34 (85) |
|  | 1–3 can of beer (2.8%, 50cl) | - | - | 1 (2.5) | 1 (2.5) | 2 (5) |
|  | 1–3 glass of wine (12–15 cl) | - | - | 2 (5) | 5 (12.5) | 4 (10) |
| Q10 | Do you use mouth-wetting agents? If yes, how often?  (e.g., food oil, gel, spray, artificial saliva substitutes, etc.) |  |  |  |  |  |
|  | Several times a day (more than three times) | 5 (12.5) | 3 (7.5) | 3 (7.5) | 2 (5) | 3 (7.5) |
|  | Three times a day | 3 (7.5) | 4 (10) | 4 (10) | 4 (10) | 3 (7.5) |
|  | Once or twice a day | 6 (15) | 6 (15) | 4 (10) | 2 (5) | 1 (2.5) |
|  | 4–6 times a week | 1 (2.5) | - | 3 (7.5) | 4 (10) | 3 (7.5) |
|  | 1–3 times a week | 5 (12.5) | 5 (12.5) | 5 (12.5) | 3 (7.5) | 4 (10) |
|  | 1–3 times a month | - | 2 (5) | 2 (5) | - | - |
|  | Never | 20 (50) | 20 (50) | 19 (47.5) | 25 (62.5) | 26 (65) |
| Q11 | Do you use sugar-free chewing gum or sucking tablets  (e.g., xylitol, fluoride, apple acid, and citric acid)? |  |  |  |  |  |
|  | Several times a day (more than three times) | 4 (10) | 5 (12.5) | 6 (15) | 2 (5) | 3 (7.5) |
|  | Three times or more a day | 4 (10) | 3 (7.5) | 3 (7.5) | 3 (7.5) | 1 (2.5) |
|  | Once or twice a day | 4 (10) | 4 (10) | 2 (5) | 3 (7.5) | 4 (10) |
|  | 4–6 times a week | 10 (25) | 1 (2.5) | 3 (7.5) | 2 (5) | 2 (5) |
|  | 1–3 times a week | 7 (17.5) | 11 (27.5) | 10 (25) | 7 (17.5) | 5 (12.5) |
|  | 1–3 times a month | 6 (15) | 5 (12.5) | 3 (7.5) | 10 (25) | 8 (20) |
|  | Never | 5 (12.5) | 11 (27.5) | 13 (32.5) | 13 (32.5) | 17 (42.5) |
| Q12 | Do you frequently sip water/fluid, adequately intake  water/fluid or fluid food (soup, gravy, yogurt, etc.)? |  |  |  |  |  |
|  | Several times a day (more than three times) | 30 (75) | 27 (67.5) | 28 (70) | 25 (62.5) | 21 (52.5) |
|  | Three times a day | 10 (25) | 13 (32.5) | 12 (30) | 15 (37.5) | 19 (47.5) |
|  | Once or twice a day | - | - | - | - | - |
|  | 4­–6 times a week | - | - | - | - | - |
|  | 1–3 times a week | - | - | - | - | - |
|  | 1–3 times a month | - | - | - | - | - |
|  | Never | - | - | - | - | - |
| Q13 | Have you decreased the use of sweet foods and soft drinks?  (e.g., Coca Cola, candy, beverages with sugar, sweet cake) |  |  |  |  |  |
|  | always | 31 (77.5) | 29 (72.5) | 26 (65) | 25 (62.5) | 26 (65) |
|  | most of times | 9 (22.5) | 8 (20) | 11 (27.5) | 9 (22.5) | 4 (10) |
|  | often | - | - | - | - | 3 (7.5) |
|  | sometimes | 0 (0) | 3 (7.5) | 3 (7.5) | 6 (15) | 7 (17.5) |
|  | Never | - | - | - | - | - |
| Q14 | Do you avoid using irritating agents for oral health  (e.g., spicy food or hard toothbrush)? | - | - | - | - | - |
|  | always | 23 (57.5) | 20 (50) | 19 (47.5) | 15 (37.5) | 14 (35) |
|  | most of times | 9 (22.5) | 14 (35) | 15 (37.5) | 11 (27.5) | 12 (30) |
|  | often | - | - | - | 5 (12.5) | 5 (12.5) |
|  | sometimes | 8 (20) | 6 (15) | 6 (15) | 9 (22.5) | 9 (22.5) |
|  | Never | - | - | - | - | - |
| Q15 | Do you use of air humidifier at night? |  |  |  |  |  |
|  | always | 16 (40) | 18 (45) | 14 (35) | 13 (32.5) | 15 (37.5) |
|  | most of times | 6 (15) | 3 (7.5) | 5 (12.5) | 6 (15) | 8 (20) |
|  | often | 8 (20) | 4 (10) | 5 (12.5) | 6 (15) | 6 (15) |
|  | sometimes | 10 (25) | 15 (37.5) | 16 (40) | 13 (32.5) | 8 (20) |
|  | Never | - | - | - | 2 (5) | 3 (7.5) |
